# From Estimated Targets to Verified Coverage: Implementation of a Community Health Worker-Based Tracking Intervention to Address Denominator Inaccuracies in High-Risk Urban Settings of Balochistan, Pakistan

**DOI:** 10.3390/vaccines14030262

**Published:** 2026-03-13

**Authors:** Rubab Kamran, Maliha Fatima, Babar Shahid, Muhammad Ayaz, Farid Ullah Khan, Simran Siraj, Zainab Farid, Noshad Ali, Ali Turab, Soofia Yunus, Zaffar Iqbal, M. Imran Khan

**Affiliations:** 1 Precision Health Consultants (PHC) Global (Private) Limited, Karachi 74800, Pakistan; rubab.kamran@phcglobal.org (R.K.); maliha.fatima@phcglobal.org (M.F.); muhammad.ayaz@phcglobal.org (M.A.); farid.ullah@phcglobal.org (F.U.K.); simran.siraj@phcglobal.org (S.S.); zainab.farid@phcglobal.org (Z.F.); noshad@phcglobal.org (N.A.); ali@phcglobal.org (A.T.); 2 Health Department, Government of Balochistan, Quetta 87100, Pakistan; drbabarshahid@gmail.com (B.S.); zaffarkhosti415@gmail.com (Z.I.); 3 Federal Directorate of Immunization Government of Pakistan, Islamabad 44000, Pakistan; soofiayunus@yahoo.com

**Keywords:** immunization coverage, zero-dose children, community health workers, CHW books, routine immunization, operational integration

## Abstract

**Background**: Routine immunization programs in Pakistan rely heavily on estimated population denominators, limiting accurate identification and follow-up of zero-dose and under-immunized children, particularly in high-risk urban settings such as Quetta, Balochistan. **Methods**: A quasi-experimental, pre–post implementation study was conducted from June 2024 to June 2025 across three low-performing union councils. A CHW-based household tracking tool was integrated within existing PEI–EPI systems. Data were derived from CHW Books, Rapid Convenience Assessments (RCA), and routine MIS reports. Descriptive statistical analysis was employed to assess trends in immunization coverage and program performance; no inferential statistical tests were applied due to the use of complete programmatic (census-based) data rather than sampled observations. **Results**: Antigen-specific coverage improved substantially, with Penta-3 coverage increasing from 22% to 95%, zero-dose conversion from 45% to 65%, and defaulter follow-up from 14% to 79%. All three union councils transitioned to Category 1 operational status. Administrative coverage exceeding 100%, reflecting population-level coverage and denominator correction rather than true population-level coverage. **Conclusions**: Integrating CHW-based tracking with dynamic denominator verification enhances routine immunization, microplanning, equity, and operational performance in high-risk LMIC urban settings.

## 1. Introduction

Immunization is one of the most cost-effective public health interventions, preventing an estimated 4–5 million deaths worldwide annually [1,2,3]. Despite decades of progress, gaps in coverage persist, particularly in low- and middle-income countries (LMICs), where millions of children remain under-immunized or completely unvaccinated [2]. In 2023, an estimated 14.3 million children worldwide did not receive a single dose of the diphtheria–tetanus–pertussis (DTP1) vaccine and were classified as zero-dose (ZD) children (UNICEF & WHO, 2024) [2]. These children are disproportionately concentrated in marginalized, conflict-affected, or underserved areas, highlighting persistent inequities in health systems.

Pakistan continues to face systemic, social, and operational barriers to routine immunization. The country accounts for nearly 396,000 zero-dose children, placing it among the top ten global contributors to the ZD burden [3,4]. Despite progress, Pakistan has not met the global benchmarks of 90% national and 80% district-level coverage [5]. The most recent Third-Party Verification of Immunization Coverage Survey (TPVICS 2020–2021) reported that only 76% of children aged 12–23 months were fully immunized. Coverage varied widely across antigens, from 93% for BCG to 82% for the third dose of pentavalent vaccine (Penta-3) and 80% for measles-1 [6,7]. Beneath these averages, provincial disparities are stark: Punjab reached 85% fully immunized coverage, whereas Balochistan lagged at just 37% [6,7]. Alarmingly, nearly 4% of Pakistani children remain zero-dose, never having received a single vaccine.

The challenge of low vaccination coverage is particularly critical in Balochistan, especially in Quetta District, where coverage is extremely low despite its urban status [8]. Peri-urban and informal settlements face high mobility, security risks, and socio-economic inequities, which hinder access and result in a disproportionate concentration of zero-dose children [9,10]. Structural barriers, including weak microplanning, fragmented service delivery, and inadequate defaulter tracking, combined with demand-side challenges such as vaccine mistrust, misinformation, and restrictions on women’s mobility, perpetuating persistent under-immunization [11,12,13,14].

Although global and regional evidence supports the integration of polio infrastructure and CHW platforms to strengthen routine immunization delivery, there remains limited empirical evidence from Pakistan addressing foundational data challenges within routine immunization systems [15,16,17]. Most integration efforts to date have focused on coordination of service delivery, with little attention to correcting denominator inaccuracies at the household level that constrain accurate identification and tracking of zero-dose and defaulter children [15,18]. In Pakistan, routine immunization coverage remains suboptimal, and administrative targets often mask underlying inequities, with significant provincial and urban–rural disparities in full immunization and data quality [17,19]. This gap is particularly pronounced in high-risk urban settings such as Quetta, where persistent zero-dose prevalence highlights the need for data-driven integration strategies that combine robust denominator verification with service delivery coordination [15,16,18].

This study evaluates a context-specific CHW-based tracking intervention, implemented within existing EPI and PEI structures, to assess its effectiveness in improving identification, follow-up, and immunization coverage of zero-dose and under-immunized children in high-risk urban settings of Quetta, Pakistan.

## 2. Methodology

### 2.1. Study Design and Setting

This study employed a prospective, quantitative, quasi-experimental pre–post intervention design conducted between June 2024 and June 2025 across three high-risk union councils (UCs)—Kotwal-2, Kuchlak-C, and UC-13C—in Quetta District, Balochistan, Pakistan. The selected UCs are characterized by persistently low routine immunization (RI) performance, high zero-dose counts, limited access to and underutilization of EPI services, and entrenched community resistance, all of which impede effective vaccine delivery.

Quetta holds significant global public health relevance, as it remains a recognized poliovirus reservoir [6] and reports one of the lowest fully immunized child (FIC) coverage rates in Pakistan (29%) [19]. The selected sites include urban slums, peri-urban settlements, and highly mobile populations, representing fragile and underserved settings where immunization programs face substantial operational challenges. These characteristics made the sites well-suited for testing operational innovations with potential applicability beyond Pakistan.

The intervention was designed and implemented in collaboration with Pakistan’s national and provincial Expanded Program on Immunization (EPI) authorities and the Polio Eradication Initiative (PEI). This collaborative model ensured that the approach was embedded within existing immunization and polio infrastructure, thereby enhancing feasibility and scalability.

### 2.2. Study Population

The study population included all children under two years of age residing in the selected UCs during the study period. The average total population of each UC was ~43,814, with estimated immunization targets of 123 children in Kotwal-2, 129 in Kuchlak-C, and 151 in 13-C.

All age-eligible children permanently residing within the catchment areas of the three union councils were made part of the study, and children who have permanently migrated out of the study area during the study period or had incomplete household verification records were not made part of the study.

A census-based enrolment strategy was employed using community health workers (CHW) (therefore, no formal sample size or statistical power calculation was performed), which were used as a primary tool to document vaccination status, including zero-dose children, defaulter and refusal cases, and guiding follow-up until full immunization was achieved.

### 2.3. PHC Global CHW Books: Design, Operationalization, and Benchmark Intervention

#### 2.3.1. Design and Purpose of CHW Books

The intervention was designed by Precision Health Consultants (PHC) Global as a program-specific operational model to strengthen immunization system performance in underserved urban union councils of Quetta. It was informed by global normative guidance on immunization integration, including the Expanded Programme on Immunization’s emphasis on integrated service delivery and the Immunization Agenda 2030 (IA2030) strategic priorities highlighting equity, integration, and data driven microplanning [20,21,22]. 

#### 2.3.2. CHW Books and Field Deployment

Standardized Community Health Worker (CHW) Books were introduced as modified immunization tracking tools to replace fragmented registers and reliance on projected denominators. The books functioned as a real-time household census, capturing verified data on eligible children under two years of age, immunization status, zero-dose and defaulter classification, and follow-up needs. Each union council was subdivided into operational blocks consistent with the existing PEI geographic structure and assigned to CHWs to ensure accountability and eliminate duplication. Initial and refresher trainings were conducted from August through October 2024 to standardize data collection procedures and enhance tracking of zero-dose and partially vaccinated children.

#### 2.3.3. Denominator Construction and Interpretation

Household-level verification progressively expanded denominators over time, occasionally resulting in administrative coverage indicators exceeding 100%. These values reflect the correction of previously underestimated denominators and immunization of backlog cohorts, rather than true population-level coverage or program overperformance. Routine coverage recalculation based on fixed baseline denominators was therefore not methodologically appropriate due to the intentionally dynamic nature of denominator construction. RCA surveys were convenience-sampled (≥7 children per assessment per UC) and utilized caregiver recall and vaccination cards; informed consent was obtained verbally from all caregivers before participation. Accordingly, recalculation of coverage using fixed baseline denominators was not methodologically appropriate, as denominators were intentionally dynamic and refined through ongoing household verification.

#### 2.3.4. Community-Based Coverage Planning

The intervention employed a tailored, community-driven planning approach. The AS and CHWs collaborated to sensitize households, schedule outreach sessions, and mobilize caregivers, particularly in hard-to-reach populations. Data from CHW Books were used to develop targeted microplans, enabling identification of underserved clusters and systematic tracing of missed children.

### 2.4. Microplanning and Implementation

PHC Global collaborated with the EPI and PEI teams to develop evidence-informed outreach plans. Microcensus data, CHW books, UC RI records, and vaccinator registers were reviewed to identify zero-dose children, defaulters, and underserved geographic clusters. Monthly outreach session plans were co-developed, with clearly defined roles for CHWs, AS, and UC-level leadership, including UC Polio Officers (UCPOs) and UC Development Officers (UCDOs).

**Figure 1:**
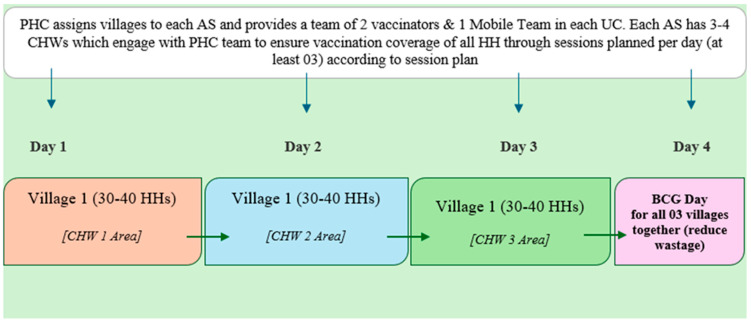
PHC Global coverage plan—community need-based, tailored approach.

Each AS was coordinated by a team comprising two vaccinators and one mobile unit, supported by three to four CHWs. Outreach sessions followed a structured three-day rotational cycle per CHW catchment, ensuring equitable coverage. PHC Global maintained 100% vaccinator availability throughout the study period, and all outreach activities were guided by regularly updated zero-dose and defaulter lists. The details are presented in Figure 1.

PHC Global did not directly administer vaccines; rather, the intervention strengthening identification, tracking, microplanning, and follow-up mechanisms, within the existing EPI service delivery system. This ensured improvement in coverage without duplication of vaccination functions.

CHWs played a central role in caregiver mobilization, household communication, and outreach site facilitation. To support accurate tracking and ensure accountability, PHC Global designed, distributed, and standardized CHW books across all UCs. These tools maintained under-two child records, identified immunization gaps, and structured monthly outreach activities.

Routine performance assessments were conducted using data from outreach sessions, Rapid Coverage Assessments (RCA), and the Monitoring Information System (MIS). This enabled real-time performance monitoring and adaptive refinement of microplans based on emerging field realities. Defaulter tracing mechanisms were strengthened, and community mobilization strategies were adjusted according to CHW feedback. The intervention focused on enhancing identification, tracking, and follow-up processes within the existing EPI service delivery system, without directly administering vaccines, ensuring improved coverage without duplication of vaccination functions.

### 2.5. Data Collection and Tools

Quantitative data were collected monthly from June 2024 to June 2025 across three target UCs of District Quetta using CHW books, Rapid Coverage Assessments (RCA), and the Monitoring Information System (MIS). RCA surveys assessed antigen-specific coverage using vaccination cards and caregiver recall, with verbal informed consent obtained from caregivers.

### 2.6. Data Analysis

Data were entered and analyzed using Microsoft Excel. Descriptive statistics (percentages, trends, and time-series comparisons) were used to assess changes in antigen-specific coverage, zero-dose conversion, defaulter follow-up, and outreach performance. Inferential statistical testing was not conducted because analyses were based on complete programmatic data rather than sampled populations.

### 2.7. Ethical Consideration

This study received approval from the AEIRC Ethics Review Committee (ERC). The consent for the study is attached to the Supplementary Files and for the RCA, verbal consent was obtained.

## 3. Results

Over the 13-month implementation period, all three intervention union councils demonstrated substantial improvements in immunization coverage, zero-dose identification, defaulter follow-up, and operational performance.

### 3.1. Rapid Convenience Assessment (RCA)

A total of 99 RCA was completed, each targeting at least seven children under the age of two through a standardized tool that captured vaccination status based on card verification and caregiver recall.

**Figure 2:**
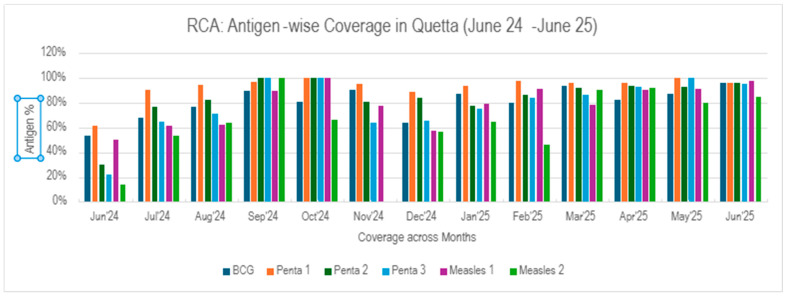
Antigen-Wise Immunization Coverage Trends Based on Rapid Convenience Assessments (June 2024–June 2025).

Consistent improvement in coverage across antigens was observed post August 2024, coinciding with the distribution of CHW immunization books and intensive training sessions for CHWs, vaccinators and area supervisors. BCG coverage increased from 54% in June 2024 to 96% in June 2025, showing a steady upward trend except for a temporary drop to 64% in December. The Penta series exhibited particularly strong gains. Penta 1 coverage rose from 62% to 100% by November 2024 and was maintained above 94% thereafter. Similarly, Penta 2 improved from 30% to 96%, and Penta 3, which initially lagged at 22%, rose consistently to reach 95% by June 2025. These trends highlight successful follow-up and timely administration of subsequent doses. Measles antigen coverage demonstrated marked improvement over the period. Measles 1 coverage increased from 50% in June 2024 to 97% in June 2025, indicating substantial progress in first-year vaccination uptake. Similarly, Measles 2 coverage improved from a baseline of 14% in June 2024 to 85% in June 2025. The trend can be seen in Figure 2 and Table 1. Though coverage declined sharply in November and December, it rebounded effectively by February and remained above 80% through the final quarter, reflecting strengthened second-year immunization efforts. Temporal alignment of performance spikes with the post-August deployment of CHW tools and capacity-building activities suggests a direct operational impact on field-level data accuracy, child tracking, and vaccination session follow-through.

### 3.2. UCs Categorization Status

These improvements in coverage are also marked by progressive shifts in the categorization status of the target UCs from January 2024 to June 2025. At baseline (Jan–May 2024), none of the three UCs were in optimal operational status: Kuchlak-C was classified as Category 4, indicating systemic deficits in both access and utilization; Kotwal-2 was in Category 3, suggesting reasonable uptake among reached populations but poor geographical or logistical access; and 13-C fell under Category 2, implying accessible services but suboptimal community utilization.

By the midline period (June–Nov 2024), targeted interventions appear to have yielded partial results. Kotwal-2 shifted to Category 1, demonstrating concurrent improvements in both dimensions. 13-C and Kuchlak-C moved to Category 2, reflecting improved access but persistent utilization gaps.

At endline (Apr–Jun 2025), all three UCs had transitioned to Category 1 status, denoting excellence in both access and utilization of immunization services. This shift from the initial distribution across Categories 2–4 to uniform placement in Category 1 represents a substantive systems-level gain resulting from effective PEI–EPI synergy through CHW books in target UCs of Quetta.

### 3.3. Fully Immunized Child (FICs)

**Figure 3:**
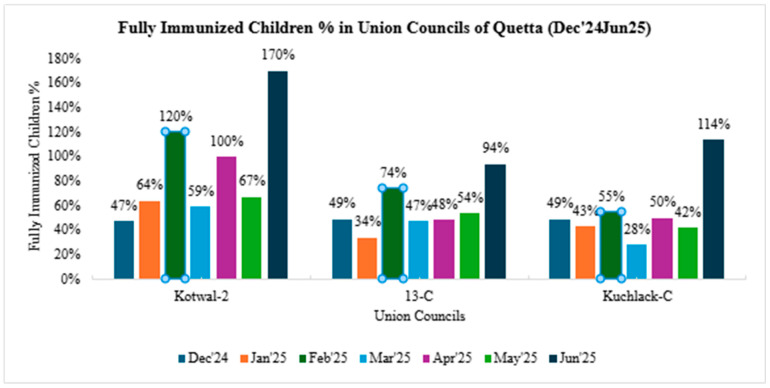
Fully immunized child percentage in selected union councils of Quetta.

Each union council demonstrated substantial immunization activity over the reporting period, with a total of 8269 children vaccinated in Kotwal-2, 7971 in 13-C, and 7957 in Kuchlak-C from June 2024 to June 2025. From December 2024 to June 2025, Fully Immunized Child (FIC) demonstrated a marked upward trend across all three union councils, both in scale and consistency. In Kotwal-2, FIC rose from 47% in December to 170% in June 2025, with a consistent trajectory of improvement observed from February onward, reflecting sustained strengthening of outreach, backlog clearance, and improved follow-up mechanisms. Similarly, 13-C advanced from 49% in December to 94% in June, maintaining a steady upward trend since March. Kuchlak-C achieved 114% FICs in June 2025 as compared to 49% in December. FIC% trends can be seen in Figure 3.

These values above 100% represent the inclusion of children who were previously uncounted in baseline estimates, demonstrating correction of underestimated denominators rather than true over-vaccination.

### 3.4. Outreach Performance

Outreach functionality was maintained to ensure effective synergy. Hence, between June 2024 and June 2025, outreach performance demonstrated sustained operational efficiency and strengthened accountability across all assessed parameters (*N* = 73 outreach sessions). Key service delivery indicators: OR opening, vaccinator availability and vaccine stock availability remained at 100% throughout the period, reflecting high adherence to outreach implementation standards.

**Figure 4:**
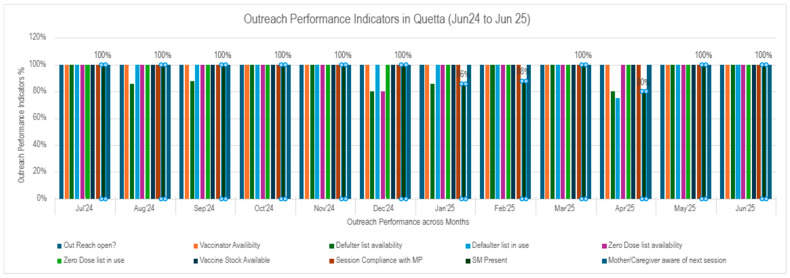
Outreach performance indicators percentage.

**Figure 5:**
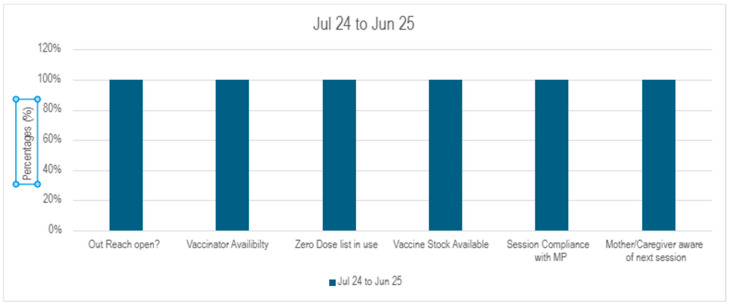
Outreach performance indicators (July 24–Jun 25). Monthly Defaulter coverage trends in intervention union councils.

A notable improvement was observed in the availability and operationalization of reporting tools as indicated by reporting tools indicators. ZD and the defaulters list availability remained 80% or above. Similarly, use of the defaulter and ZD lists was reported to be 100% throughout except dips in April 2025 and December 2024, respectively, can be seen in Figure 4 and Figure 5. All sessions followed MP throughout the reporting period

Social Mobilizer (SM) presence, while generally strong, showed periodic declines of 86% in January, 88% in February, and 80% in April, indicating room for stability in community engagement support. Despite these fluctuations, caregiver awareness of next session dates remained consistently at 100%, pointing to effective communication mechanisms.

### 3.5. Zero-Dose and Defaulters Coverage

**Figure 6:**
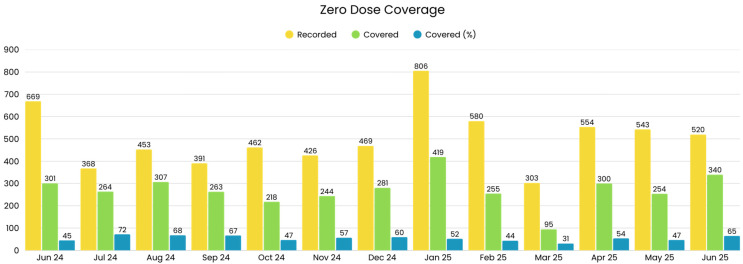
Monthly zero-dose (ZD) coverage trends in intervention union councils (June 2024–June 2025).

The analysis of zero-dose (ZD) coverage between June 2024 and June 2025 reveals marked fluctuations in identification and conversion yet demonstrates overall progress in closing immunization equity gaps. The initial ZD coverage was 45% in June 2024, gradually improving to 65% by June 2025, an absolute gain of 20 percentage points. Notably, a sustained ≥60% coverage was achieved during key months such as July (72%), August (68%), and again in December (60%) and June 2025 (65%), reflecting operational effectiveness in select periods. ZD coverage percentage can be seen in Figure 6.

**Figure 7:**
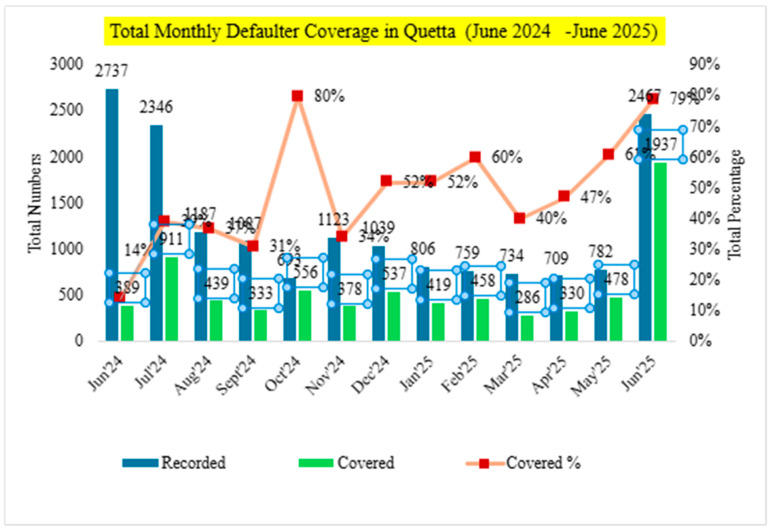
Monthly defaulter coverage trends in intervention union councils (June 2024–June 2025).

This trajectory suggests improved systematic identification, tracking, and follow-up of zero-dose children, likely facilitated by the introduction and active use of integrated ZD lists within outreach and fixed-site service delivery. However, ZD coverage declined from 67% in September to 47% in October 2024, from 52% in January to 44% in February 2025, and slightly from 54% in April to 47% in May 2025, before rebounding to 65% in June 2025. The total monthly default coverage percentage can be seen in Figure 7. These dips suggest a possible programmatic focus on recording and mobilizing ZD cases in preparation for intensified activity during BCU rounds, which may temporarily depress monthly coverage percentages due to expanded denominators or shifting resource allocation.

Similarly, defaulter coverage exhibited a substantial upward trajectory, underscoring the effectiveness of strengthened PEI–EPI synergy, particularly through the structured deployment of CHW immunization tracking books. These tools enabled more systematic identification, follow-up, and conversion of defaulters into fully immunized children, with monthly data reflecting both improved accuracy in defaulter enumeration and field responsiveness. Coverage rose from a modest 14% in June 2024 (389 of 2737) to a robust 79% by June 2025 (1937 of 2467), highlighting a nearly sixfold increase in effectiveness. Sharp improvements observed in October 2024 (80%), February 2025 (60%), and June 2025 (79%) coincide with periods of intensified outreach during BCU rounds, further validating the system’s capacity to scale during peak mobilization. However, the sustained month-on-month gains are primarily attributable to embedded field-level synergy, rather than episodic campaigns, indicating a maturing and resilient immunization delivery mechanism.

### 3.6. MIS Antigen-Wise Coverage

The year-long trend across Kotwal-2, Kuchlak-C, and W13-C reveals a decisive shift in immunization system performance, shaped by field-driven innovations and cross-programmatic synergy. From June 2024 to June 2025, all three UCs demonstrated substantial, and in many cases exponential, increases across core EPI antigens over the reporting period, underscoring the operational impact of intensified ZD and defaulter tracking mechanisms, institutionalized through the CHW books and strengthened PEI–EPI coordination.

In Kotwal-2, Penta-II coverage rose from 52% to 328%, and MR-II from 34% to 420%, reflecting targeted follow-up of previously missed children and intensified backlog immunization. Coverage surpassing 100% in these antigens illustrates the correction of undercounted denominators through continuous household verification, rather than over-reporting or administering vaccines to children beyond the eligible population.

Similarly, in Kuchlack-C, Penta-II coverage increased from 42% to 193% (↑151%), Penta-III from 37% to 189% (↑152%), MR-I coverage rose from 32% to 316% (↑284%), and MR-II from 16% to 176% (↑160%). These triple-digit percentages underscore the intervention’s ability to capture and vaccinate children not previously included in programmatic counts, providing evidence of improved denominator accuracy and operational effectiveness.

**Figure 8:**
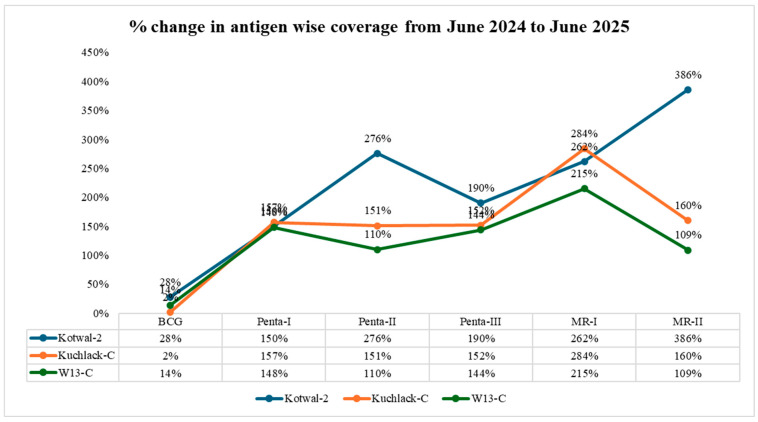
Antigen-wise coverage percentage June 2024–June 2025.

W13-C also recorded pronounced increases, notably MR-I (39% to 254%) and Penta-I (48% to 196%), demonstrating consistent upward trajectories and successful follow-up. In all instances, coverage exceeding 100% should be interpreted as a reflection of backlog clearance and improved denominator validation, not actual over-vaccination. Antigen wise coverage percentage from June 2024 to June 2025 can be seen in Figure 8.

## 4. Discussion

The study provides implementation-level evidence demonstrating that operational integration of the PEI and EPI, supported by a structured, community-based tracking system, can substantially improve routine immunization performance in high-risk, underserved urban settings. The introduction of PHC Global-designed Community Health Worker (CHW) immunization books, combined with targeted capacity-building and block-based microplanning, was associated with marked improvements in antigen-specific coverage, zero-dose identification and conversion, defaulter follow-up, and outreach functionality across all three intervention union councils in Quetta.

Unlike many conventional integration efforts that primarily emphasize service delivery coordination, this intervention addressed a foundational systemic weakness: reliance on estimated denominators and fragmented frontline data. Implementation of verified denominators through CHW Books enabled accurate identification of zero-dose and defaulter children, guiding evidence-based microplanning and real-time prioritization of outreach activities.

The operational outcomes were substantial: Measles-2 coverage, which historically reflects weak second-year service utilization, increased from 14% to 85%, while zero-dose coverage rose from 45% to 65%, and defaulter coverage improved nearly sixfold, from 14% to 79% over the study period. These gains illustrate the impact of accurate denominator data in reducing dropout between successive doses, a persistent challenge in high-mobility urban settlements. Administrative coverage occasionally exceeding 100% reflects the vaccination of previously uncounted children and backlog clearance, indicating correction of underestimated denominators rather than over-reporting or population over-vaccination.

All three union councils progressed from lower operational categories (2–4) to Category 1 status, reflecting the strengthening of both supply- and demand-side dimensions of routine immunization, including access, utilization, and community engagement. MIS data further corroborated these findings, demonstrating triple-digit percentage increases across multiple antigens, particularly in historically underperforming union councils, highlighting the system’s capacity to absorb, track, and immunize missed cohorts even in high-mobility, security-constrained urban contexts. These outcomes underscore that systematic, CHW-driven tracking mechanisms, rather than episodic campaigns alone, are essential for durable equity gains.

In terms of feasibility, the study demonstrates that a CHW-based household tracking system can be successfully integrated within existing PEI–EPI structures, evidenced by sustained implementation over 13 months, full integration with routine outreach workflows, and consistent use of CHW tools by frontline staff. Verified household-level data enabled more accurate identification of zero-dose and defaulter children, improved microplanning, and enhanced follow-up, contributing directly to the observed improvements in both coverage and UC operational categorization.

Despite these strengths, several limitations are noted. The study relied on routine administrative and programmatic data, limiting causal inference and external validation. The absence of independent coverage surveys and the use of convenience-sampled RCA may introduce reporting bias. Additionally, household-level enumeration is labor-intensive, which could pose challenges for scaling larger populations. Future scale-ups may require digital enumeration tools, task-shifting to additional field staff, or community-led verification mechanisms to maintain high-quality household-level verification. Triangulation across multiple data sources mitigated some of these limitations and supports the operational robustness of the integrated PEI–EPI model in high-risk urban contexts.

### Recommendations

Based on the findings from this study, as well as the experience with the integrated service delivery for PEI–EPI, the following recommendations have been made with the aim of improving the performance of Routine Immunization, reducing the burden of zero doses, as well as enhancing the efficiency of the system in the province of Balochistan:

i. Scaling up of Integrated PEI–EPI Delivery Model

The integrated immunization delivery platform based on the PEI operational geographic framework (AS Blocks and CHW Sub-blocks) needs to be expanded to more Union Councils at high risk of poor routine immunization coverage and elevated prevalence of zero doses. Building what already exists in the PEI infrastructure, resources, and microplanning structures may optimize outreach efforts.

Formalization of Block-Based Microplanning Routine Immunization Microplans need to be harmonized on the predetermined block and sub-block division of PEI. Vaccinators in each Union Council must retain equal numbers of AS Block allocations to maintain a focus of accountability, sustained delivery, and total area coverage in the monthly outreach program.

ii. Standardization & Digitization of CHW Immunization Register

Immunization registers maintained by CHWs at the household level should ideally be standardized at the district level, with progressive computerization if possible. Immunization registers should be formally acknowledged as important data sources for identifying persons with no doses, defaulters, or planning outreach immunization sessions.

iii. Institutionalization of Joint PEI–EPI Supervision

Regular joint supervisory visits, including staff members of PEI and EPI, must be made at the Union Council and facility levels. A checklist for supervision can also be utilized to ensure the verification of microplans, confirmation of coverage data, and evaluation of readiness in the cold chain/logistical sector. Integration of Low-Cost Coverage Verification Mechanisms. There is a need for regular use of Lot Quality Assurance Sampling (LQAS) assessments and other rapid measurement tools to be included in the districts’ monitoring plans to supplement the data collected in the management information system. This would help in the early detection of areas under coverage and errors in the denominator in the mobile and peri-urban areas.

iv. Enhancing Community Engagement via Social Mobilization

Social mobilizers need to be incorporated at a block and sub-block level within the EPI microplan, and their roles related to community mobilization, refusal handling, follow-up of defaulters, and support during sessions need to be clearly demarcated. Orientation, monitoring, and feedback systems need to be developed to make these workers effective.

v. Integration of Vaccine-Preventable Disease (VPD)

The current structure of the AS-CHW should be improved beyond immunization sessions to cover the surveillance of priority VPDs such as measles, AFP, NT, DT, and other/notifiable conditions through the communities. Training of CHWs should be conducted on case definitions, while the ASs and vaccinators should verify the process for completeness, while taking into consideration the gaps between immunization sessions and the occurrence of the diseases.

vi. Post-Polio Transition Care Support

Health System Strengthening: Such a model of PEI integration within the block structure could also play a role in post-polio transition planning by ensuring the retention of PEI assets within the framework of overall public health functions for the betterment of primary health care.

## 5. Conclusions

Overall, this study demonstrates that a CHW-based, denominator-correcting tracking intervention, embedded within existing PEI and EPI structures, can transform routine immunization performance into high-risk urban settings. By converting estimated targets into verified household-level coverage data, the intervention enables targeted, efficient, and equity-driven immunization delivery, providing actionable evidence for policymakers and program managers seeking scalable, low-cost solutions to address persistent immunization gaps in LMIC urban contexts.

## Figures and Tables

**Table 1:** Antigen-specific coverage at baseline and endline (RCA Data) (month-wise details (13 months data) for all the antigens are present in the Supplementary Materials).

Antigen	June 2024 (%)	June 2025 (%)	Absolute Change (Percentage Points)	Relative Increase (%)
BCG	54	96	+42	+77.8
Penta-1	62	96	+34	+54.8
Penta-2	30	96	+66	+220
Penta-3	22	95	+73	+332
Measles-1	50	97	+47	+94
Measles-2	14	85	+71	+507

Note: RCA conducted monthly across three intervention union councils. Absolute change calculated as June 2025 minus June 2024.

## Data Availability

Data are contained within the article.

## Supplementary Materials

The following supporting information can be downloaded at: https://www.mdpi.com/article/10.3390/vaccines14030262/s1, Table S1: Rapid Convenience Assessment (RCA): Baseline vs Endline Coverage Antigen-Specific Coverage at Baseline and Endline (RCA Data).
