# Supplementary material for: From Estimated Targets to Verified Coverage: Implementation of a Community Health Worker-Based Tracking Intervention to Address Denominator Inaccuracies in High-Risk Urban Settings of Balochistan, Pakistan
Source: Vaccines (Basel). 2026 Mar 13;14(3):262. doi: 10.3390/vaccines14030262 (PMC13029881; doi:10.3390/vaccines14030262)
Supplement: Supplementary file 1 [file vaccines-14-00262-s001.zip › vaccines-4132655-supplementary.pdf]

**SUPLEMENTARY TABLE:**

**Table S1: Rapid Convenience Assessment (RCA): Baseline vs Endline Coverage Antigen-Specific Coverage at Baseline and Endline (RCA Data)**

| | June 2024-Dec 2024 | | | | | | | Jan 2025-June 2025 | | | | | |
|---|---|---|---|---|---|---|---|---|---|---|---|---|---|
| Antigen/vaccine | Jun | Jul | Aug | Sep | Oct | Nov | Dec | Jan | Feb | Mar | Apr | May | Jun |
| BCG | 54% | 68% | 77% | 90% | 81% | 90% | 64% | 87% | 80% | 94% | 82% | 88% | 96% |
| Penta 1 | 62% | 91% | 94% | 97% | 100% | 95% | 89% | 94% | 98% | 96% | 96% | 100% | 96% |
| Penta 2 | 30% | 77% | 83% | 100% | 100% | 81% | 84% | 78% | 86% | 93% | 94% | 93% | 96% |
| Penta 3 | 22% | 65% | 71% | 100% | 100% | 64% | 66% | 75% | 84% | 86% | 93% | 100% | 95% |
| Measles 1 | 50% | 62% | 63% | 90% | 100% | 78% | 57% | 79% | 92% | 78% | 91% | 92% | 97% |
| Measles 2 | 14% | 53% | 64% | 100% | 67% | 0% | 57% | 65% | 46% | 91% | 92% | 80% | 85% |
